# Deaths following illicit ketamine use in England, Wales and Northern Ireland 1999–2024: An update report to inform the reclassification debate

**DOI:** 10.1177/02698811251373058

**Published:** 2025-09-29

**Authors:** Jade Pullen, John M. Corkery, Rebecca McKnight, Caroline S. Copeland

**Affiliations:** 1 National Programme on Substance Use Mortality, London, UK; 2 Hertfordshire Medical School, University of Hertfordshire, UK; 3 Manchester Metropolitan University, UK; 4 Institute of Pharmaceutical Science, King’s College London, UK

**Keywords:** ketamine, polydrug use, drug-related deaths, public health policy, drug policy

## Abstract

**Background:**

Ketamine is increasingly used in the United Kingdom in non-clinical settings for its psychoactive effects, with rising reports of harms including hospital admissions, dependence and deaths. In light of current debates surrounding the reclassification of ketamine under the Misuse of Drugs Act 1971, up-to-date surveillance of associated mortality is warranted.

**Aims:**

We aimed to quantify trends in deaths following illicit ketamine use in England, Wales and Northern Ireland, and to examine changes in demographic and contextual characteristics since the last national analysis which comprised illicit ketamine deaths up to 2019.

**Methods:**

Cases where illicit ketamine was detected at post-mortem were extracted from the National Programme on Substance Use Mortality and analysed.

**Results:**

There were 696 deaths identified with illicit ketamine between 1999 and 2024. Annual deaths increased over 10-fold from 2014 (15 deaths) to 2024 (197 projected deaths). Whilst absolute deaths implicating illicit ketamine rose (2014: 6 deaths; 2023: 123 projected deaths), the proportion of deaths where illicit ketamine was implicated in causing death declined (2014: 60.0% of cases; 2024: 42.6% of cases). Concurrently, polydrug use increased (median number of co-administered substances 1999–2004: 3; 2005–2009: 3, 2010–2014: 4, 2015–2019: 6; 2020–2024: 6), and the demographic profile of decedents shifted towards greater deprivation and dependence-related contexts.

**Discussion and Conclusions:**

There has been an acceleration in deaths following illicit ketamine in recent years, which are increasingly featuring complex patterns of polydrug use and socio-economic vulnerability. Policy responses must extend beyond single-substance legislative controls to encompass harm reduction, treatment integration, and social support strategies.

## Introduction

Ketamine has legitimate uses as an analgesic and anaesthetic in both clinical and veterinary settings (Corkery et al., 2021), with its S-enantiomer esketamine licensed for acute treatment of treatment-resistant depression (European Medicines Agency, 2025) in some countries, including the United Kingdom – although here it is only available via private prescription and not via the National Health Service. It does also, however, possess dissociative, stimulant and hallucinogenic properties (Wolff and Winstock, 2006). People seeking these effects have driven increased availability and prevalence of illicit ketamine in the United Kingdom in recent years: organised crime gangs are reportedly exploiting legal supply chains by using front companies to import ketamine – primarily from India but also China and Pakistan – for supposed medical use, then diverting it to illicit markets in Europe (EUDA, 2024; Kenyon and Grant, 2025); it is comparatively low-cost in comparison to other substances such as 3,4-methylenedioxymethamphetamine (MDMA) and cocaine (Hamilton and Sumnall, 2024) and is easily accessible both on the streets and online from social media platforms and the ‘dark web’ (Dieth and Collins, 2025); the number of people using illicit ketamine in the United Kingdom has continued to rise with an estimated 299,000 people aged 16–59 reporting illicit ketamine use in 2024 – the largest on record (Home Office, 2025a) – with waste water analysis indicating an 85% increase in ketamine consumption between 2023 and 2024 (Home Office, 2025b). The rising prevalence of illicit ketamine use is also evident globally, with survey data indicating increased use among young people in the Netherlands, Denmark, Romania and the United States (EUDA, 2023; Palamar, 2025), and wastewater analyses revealing heightened consumption in Australia, Spain, Italy, Denmark and Portugal (Australian Criminal Intelligence Commission, 2024; EUDA, 2023). Concomitantly, ketamine-related harms – which encompass physical, psychological and social harms – have also risen as evidenced in the United Kingdom by increasing hospital admissions, dependence, and deaths (Corkery et al., 2021; Office for Health Improvement and Disparaties, 2023). Ketamine was first classified by the U.K. Government as a Class C substance in 2006 under the Misuse of Drugs Act 1971, which was then upgraded to Class B in 2014. The ongoing trends of rising prevalence, use, and harms has now prompted new policy discussions to consider further escalation of ketamine controls by reclassifying it as a Class A substance (Johnson and Home Office, 2025).

A comprehensive analysis of deaths following illicit ketamine use in England was recently carried out by Corkery et al. (2021), who examined deaths from 1997 to 2019. They reported significant increases in deaths following illicit ketamine use over the prior decade and identified regional variations and specific demographic risk factors. Preliminary reports since its publication suggest that deaths following illicit ketamine use have not only continued but also accelerated (Office for National Statistics (ONS), 2025).

In this study, we present an update on deaths following illicit ketamine use in England, Wales and Northern Ireland between 2020 and 2024. By building upon the work presented in Corkery et al. (2021), we aim to quantify the extent to which illicit ketamine use is leading to death – both when used as a sole substance and when used in combination with others – and to evaluate whether previously identified demographic trends have continued or changed. Given the current political and policy interest in the harms posed by illicit ketamine – particularly in respect of current discussions of a potential reclassification to Class A – up-to-date surveillance of deaths following its use is essential. In addition to informing the reclassification debate, the data presented here will also be of use in the design and implementation of targeted harm reduction, clinical guidance, and public health messaging to mitigate illicit ketamine-related harms.

## Methods

### National Programme on Substance Use Mortality

Data were collated from case reports submitted to the National Programme on Substance Use Mortality (NPSUM; formerly the National Programme on Substance Abuse Deaths), which has received regular voluntary reports regarding deaths following drug use from coroners since 1997. Cases include deaths from prescription medications, recreational drugs, novel psychoactive substances and intravenous drug use. Coroners investigate deaths resulting from a range of causes deemed to be unnatural; this includes violent and sudden deaths, unexplained deaths, deaths that occur before a patient comes out of anaesthetic, and deaths caused by industrial disease or poisoning. Toxicology tests are requested dependent upon individual case circumstances and at the discretion of the coroner and/or consulting pathologist.

### Case identification

Cases were identified from those reported to the NPSUM by 1 March 2025 with toxicology evidence confirming the presence of illicit ketamine in decedents’ post-mortem tissue(s), in accordance with the case identification procedure followed in Corkery et al. (2021).

### Case analysis

#### Software

IBM^®^ SPSS software (Version 29) was used for case extraction, analysis and statistical tests. Microsoft Excel 365 was used for data visualisation.

#### Source of ketamine

Ketamine was classified as illicit in source where it had not been administered as part of emergency medical treatment. This information was derived from the toxicology reports as medical use of ketamine (and other drugs administered as part of medical treatment) is routinely notified to toxicologists to aid in their interpretation of the toxicological findings.

#### 2024 projection

The average time between death and coronial inquest conclusion for a drug-related death reported to the NPSUM is 7–10 months, but can be longer. Further deaths occurring in 2024 are therefore anticipated to be reported to the NPSUM after the date of the data cut made for this analysis (1 March 2025). Based on jurisdiction reporting trends, the total number of deaths occurring in 2024 expected to be received by the NPSUM has been projected.

#### Implication of illicit ketamine in causing death

Illicit ketamine was deemed to have been implicated in causing death in cases where it was explicitly listed as a cause of death by the coroner, or in cases where ambiguous drug-related causes were listed (e.g., multidrug toxicity, polydrug use) and the consulting toxicologist cited illicit ketamine use as a likely contributor in causing death. Deaths where illicit ketamine was detected but not deemed contributory in causing death are herein referred to as ‘incidental illicit ketamine deaths’ and those where ketamine was deemed caused as ‘illicit ketamine-related deaths’.

#### Ketamine use context

The context of illicit ketamine use was determined from the narrative circumstances of death. Cases deemed recreational were those where the use took place in a party/pub/bar/club/rave/music festival context. Cases deemed dependent were those where a ketamine dependency was stated or where a compulsion to use ketamine was described.

#### Indices of multiple deprivation

Deprivation quintiles (quintile 1 – most deprived; quintile 5 – least deprived) were determined by postcode matching the usual address of decedents with the English (Ministry of Housing, Communities and Local Government, 2019), Welsh (Data Cymru, 2025) and Northern Irish (Northern Ireland Statistics and Research Agency, 2025) Indices of Deprivation calculators.

#### Statistics

Chi-square tests were used to determine if associations between two categorical variables were significantly different (e.g., proportion of males and females who died 1999–2019 vs 2020–2024) and an independent two-sample Student’s *t*-test was performed to compare the mean ages of those who died following illicit ketamine use between 1999 and 2019 versus 2020–2024. The significance threshold for all tests was set at *p* < 0.05.

### Ethics

The King’s College London Biomedical and Health Sciences, Dentistry, Medicine and Natural and Mathematical Sciences Research Ethics Sub-Committee reconfirmed in August 2025 that NPSUM does not require research ethics committee review as all subjects are deceased.

## Results

### Post-mortem detections of illicit ketamine

**Figure 1.**
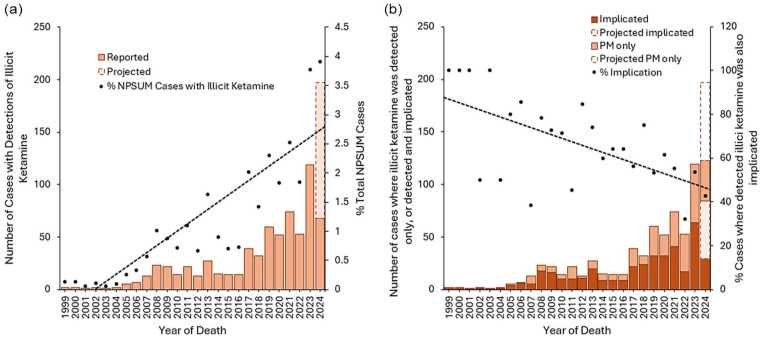
(a) All deaths reported to the NPSUM from England, Wales and Northern Ireland where illicit ketamine was detected at post-mortem, and proportion of all deaths reported to the NPSUM with illicit ketamine detected. (b) Number of incidental illicit ketamine, and illicit ketamine-related deaths, and proportion of all deaths with implications. As inquests take on average 13 months to be concluded, more deaths are expected to be received from 2024 and accordingly have been projected based upon previous trends in reporting. NPSUM: National Programme on Substance Use Mortality; PM: post-mortem.

A total of 696 deaths with post-mortem detections of illicit ketamine were identified from the NPSUM between 1999 and 2024, 363 which occurred 1999–2019 and 333 which occurred 2020–2024 (notes: no deaths were reported which occurred in 1997/8 or 2025; we have presented all following data as the year of death as opposed to the year of reporting to the NPSUM). Annual deaths are projected to have increased over 10-fold over the past decade (2014: 15 deaths; 2024: 197 projected deaths; Figure 1(a)).

### Implication of illicit ketamine in deaths

**Figure 2.**
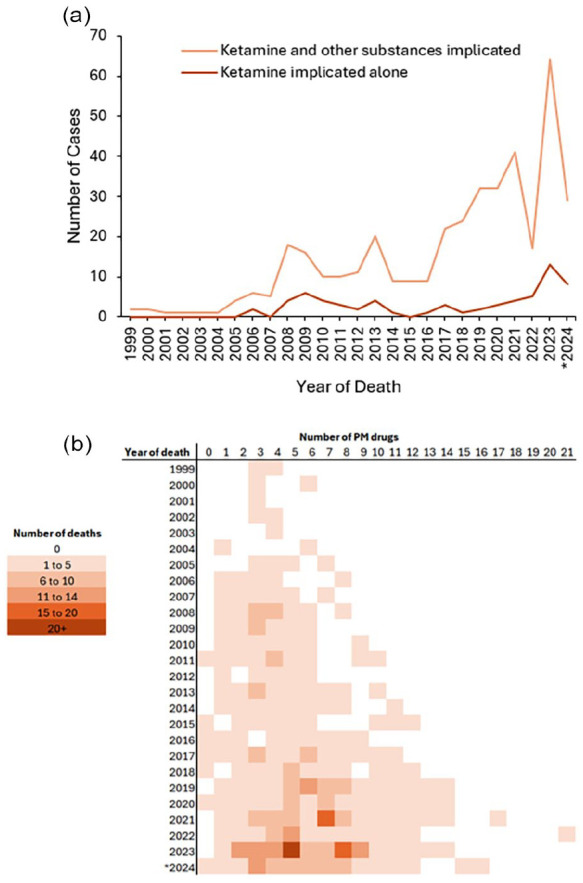
(a) Number of deaths where illicit ketamine was implicated alone or implicated in combination with other substances. (b) Number of substances detected at post-mortem in deaths with detections of illicit ketamine. *2024 data incomplete. PM: post-mortem.

Whilst the raw number of illicit ketamine-related deaths are projected to have increased 20-fold over the last decade (2014: 6 deaths; 2023: 123 projected deaths), the proportion of total illicit ketamine deaths where the illicit ketamine was implicated has decreased (2014: 60.0% of cases, *n* = 9/15; 2024: 42.6% of cases, *n* = 29/68; Figure 1(b)). At the same time, the incidence and nature of polydrug use in these deaths increased: illicit ketamine-related deaths increasingly had other co-administered substances co-implicated in causing death (Figure 2(a)), and the number of co-administered substances has also increased (median number of co-administered substances 1999–2004: 3; 2005–2009: 3, 2010–2014: 4, 2015–2019: 6; 2020–2024: 6; Figure 2(b)). In illicit ketamine-related (*n* = 396), the most commonly co-implicated substances were stimulants (45.5% of cases, *n* = 180/396) – namely cocaine (30.6% of cases, *n* = 121/396), followed by opioids (38.9% of cases, *n* = 154/396) – namely heroin/morphine (22.0% of cases, *n* = 87/396), benzodiazepines (31.6% of cases, *n* = 125/396) – namely diazepam (19.4% of cases, *n* = 77/396), and alcohol (28.5% of cases, *n* = 113/396), with no significant changes in these prevalences over time (χ^2^ *p* > 0.05 for all these substance classes 1999–2019 vs 2020–2024; Table 1). Whilst overall co-implication of gabapentinoids was low (10.1% of cases, *n* = 40/396), there was a significant increase in their co-implication over time (χ^2^ *p* < 0.05 1999–2019 vs 2020–2024; Table 1).

**Table 1.** Substance classes implicated in death following illicit ketamine use.

Substance Class Implicated	Illicit ketamine and other substances implicated						Incidental illicit ketamine with other substances implicated					
	1999–2019		2020–2024		Total		1999–2019		2020–2024		Total	
	*n*	%	*n*	%	*n*	%	*n*	%	*n*	%	*n*	%
Total	246	100	150	100	396	100	117	100	183	100	300	100
With opioid(s)	84	46.7	70	46.7	154	46.7	57	48.7	118	64.5	175	58.3
Heroin/morphine	53	29.4	34	22.7	87	22.0	45	38.5	72	39.3	117	39.0
With stimulant(s)	94	52.2	86	57.3	180	54.5	44	37.6	81	44.3	125	41.7
Cocaine	54	30.0	67	44.7	121	30.6	27	23.1	67	36.6	94	31.3
With benzodiazepine(s)	65	36.1	60	40.0	125	37.9	16	13.7	46	25.1	62	20.7
Diazepam	46	25.6	31	20.7	77	19.4	6	5.1	26	14.2	32	10.7
With alcohol	67	37.2	46	30.7	113	34.2	18	15.4	17	9.3	35	11.7
With gabapentinoids	9	5.0	31	20.7	40	12.1	6	5.1	39	21.3	45	15.0

In incidental illicit ketamine deaths (*n* = 300), the substances which were most commonly implicated were opioids (58.3% of cases, *n* = 175/300) – again namely heroin/morphine (39.0% of cases, *n* = 117/300), followed by stimulants (41.7% of cases, *n* = 125/300) – again namely cocaine (31.3% of cases, *n* = 94/300), benzodiazepines (20.7% of cases, *n* = 62/300) – again namely diazepam (10.7% of cases, *n* = 32/300), and gabapentinoids (15.0% of cases, *n* = 45/300). When comparing 1999–2019 to 2020–2024 trends, there were significant increases in the implications of the sedative drug classes: opioids, benzodiazepines and gabapentinoids (χ^2^ *p* < 0.05 1999–2019 vs 2020–2024; Table 1).

### Context of illicit ketamine use

Death was deemed accidental in the majority of cases (88.9% of cases, *n* = 619/696), with small proportions determined as suicidal (5.9% of cases, *n* = 41/696) or natural (1.1% of cases, *n* = 8/696) in nature, or of undetermined intent (4.0% of cases, *n* = 28/696). In deaths concluded as accidental in nature, the context in which the illicit ketamine was used was stated in 165 cases. Over time, the proportion of deaths which occurred in a recreational use context declined (1999–2004: 75% of cases, *n* = 3/4; 2005–2009: 69.2% of cases, *n* = 18/26; 2010–2014 50.0% of cases, *n* = 11/22; 2015–2019 38.9% of cases *n* = 14/36; 2020–2024: 28.6% of cases, *n* = 22/77) as those which occurred in a dependent use context increased. The underlying cause of death in the majority of cases was acute drug use (Table 2), with no significant changes over time in the proportion of deaths due to drug use, physiological complications, or external factors (all χ^2^ *p* > 0.05 1999–2019 vs 2020–2024; Table 2).

**Table 2.** Underlying cause in deaths following illicit ketamine use.

Underlying cause of death	1999–2019		2020–2024		Total	
	*n*	%	*n*	%	*n*	%
Total	330	100	366	100	696	100
Drug use						
Acute	276	83.6	324	88.5	600	86.2
Chronic	2	0.6	9	2.5	11	1.6
Physiological complications						
Cardiac	5	1.5	15	4.1	20	2.9
Respiratory	7	2.1	10	2.7	17	2.4
CNS	3	0.9	2	0.5	5	0.7
Urinary	1	0.3	5	1.4	6	0.9
Other	3	0.9	7	1.9	10	1.4
External factors						
Asphyxia	16	4.8	15	4.1	31	4.5
Trauma	14	4.2	8	2.2	22	3.2
Drowning	15	4.5	5	1.4	20	2.9
Unascertained	3	0.9	2	0.5	5	0.7

In some cases, multiple underlying causes were cited so the total number of causes will sum to greater than the total number of deaths.

CNS: central nervous system.

### Decedent demographics

**Figure 3.**
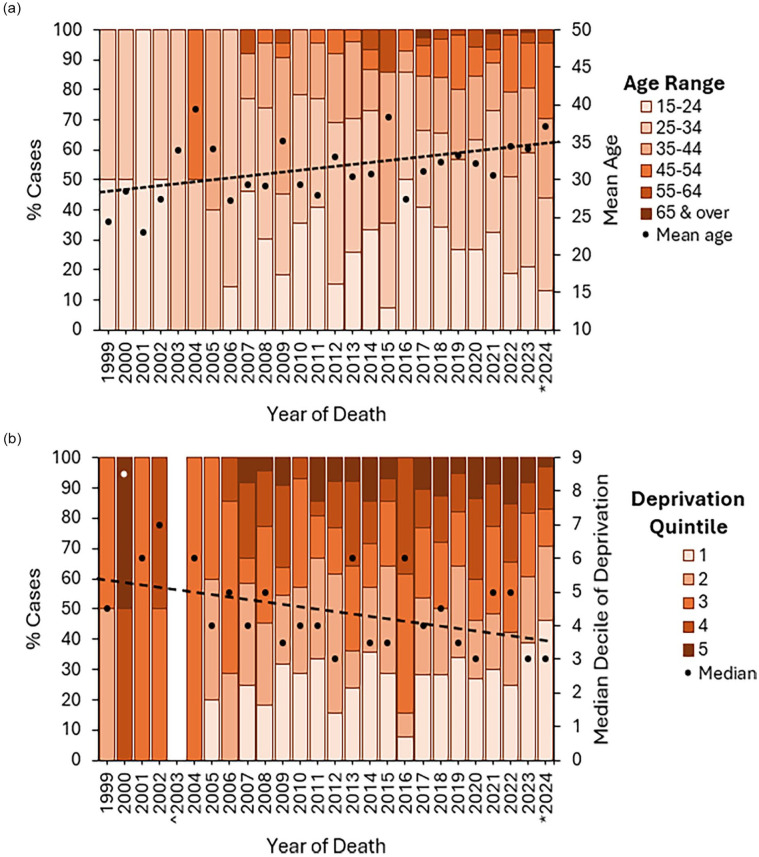
(a) Age range and mean age over time of decedents. (b) Quintile of deprivation and median quintile over time of decedents’ usual address. *2024 data incomplete. ^No deaths reported in 2003.

Since the publication by Corkery et al. (2021) examining deaths following illicit ketamine, the age at which people died has increased significantly (1999–2019: 31.5 ± 10.1, 2020–2024: 33.7 ± 11.0, *p* < 0.05; Figure 3(a)), although this still remains well below the NPSUM average (age at death 40.86 ± 13.4; Table 3). White males living with others continued to predominate illicit ketamine deaths (Table 3); however, the proportion of decedents in employment at the time of their death significantly decreased in 2020–2024 (1999–2019: 53.9% of cases, *n* = 159/295; 2020–2024: 41.5%, *n* = 117/282; χ^2^ *p* < 0.05; Table 3) trending towards the NPSUM average (30.5%), whilst the proportion living in the most deprived areas of the countries increased (Quintile 1 1999–2019: 26.2% of cases, *n* = 83/317; 2020–2024: 33.9% of cases, *n* = 124/358; Figure 3(b) and Table 3) also trending towards the NPSUM average (47.6%). A significantly greater proportion of decedents in 2020–2024 were known drug users (1999–2019 79.4% of cases, *n* = 185/233; 2020–2024 91.2% of cases, *n* = 236/258; χ^2^ *p* < 0.05; Table 3). The proportions of decedents who died in England, Wales and Northern Ireland are comparable to the NPSUM averages (χ^2^ *p* > 0.05; Table 3).

**Table 3.** Demographics of decedents who died following illicit ketamine use.

Demographic variable	1999–2019			2020–2024			All NPSUM
	*n*	%	Valid %	*n*	%	Valid %	Valid %
Total	330	100	100	366	100	100	—
Age, mean ± SD (range)	31.5 ± 10.1 (15–67)			33.7 ± 11.0 (16–68)			40.86 ± 13.4 (0–105)
Gender							
Male	273	82.7	82.7	312	85.2	85.2	73.2
Female	57	17.3	17.3	54	14.8	14.8	26.8
Ethnicity							
White	230	69.7	96.6	200	54.6	97.1	96.1
Black	2	0.6	0.8	3	0.8	1.5	1.4
Asian	5	1.5	2.1	3	0.8	1.5	1
Other	1	0.3	0.4	—	—	—	1.6
Not known	92	27.9	—	160	43.7	—	—
Employment status							
Employed	159	48.2	53.9	117	32.0	41.5	30.5
Unemployed	92	27.9	31.2	117	32.0	41.5	54.4
Homemaker	4	1.2	1.4	1	0.3	0.4	1.6
Student/pupil	33	10.0	11.2	31	8.5	11.0	2.1
Retired/long-term sick	7	2.1	2.4	16	4.4	5.7	11.4
Not known	35	10.6	—	84	23.0	—	—
Living arrangements							
Alone	97	29.4	35.9	92	25.1	38.0	46.4
With others	160	48.5	59.3	131	35.8	54.1	41.3
No fixed abode	7	2.1	2.6	7	1.9	2.9	4.5
Hostel	3	0.9	1.1	3	0.8	1.2	2.6
Student accommodation	3	0.9	1.1	7	1.9	2.9	0.2
Hotel	—	—	—	2	0.5	0.8	0.3
Other	—	—	—	—	—	—	4.7
Not known	60	18.2	—	124	33.9	—	—
Index of multiple deprivation quintile^a^							
1	83	26.2	26.2	124	33.9	33.9	47.6
2	83	26.2	26.2	74	20.2	20.2	21.3
3	74	23.3	23.3	72	19.7	19.7	14.9
4	52	16.4	16.4	55	15.0	15.0	9.4
5	25	7.9	7.9	33	9.0	9.0	6.8
Country of death							
England	309	97.5	30.7	331	90.4	90.4	86.2
Wales	11	3.5	1.1	23	6.3	6.3	3.7
Northern Ireland	9	2.8	0.9	12	3.3	3.3	3.9
Isle of Man	1	0.3	0.1	—	—	—	0.2
Scotland	—	—	—	—	—	—	5.9
Channel Islands	—	—	—	—	—	—	0.1
Known drug user							
Yes (IV)	185 (33)	56.1 (17.8)	79.4 (17.8)	236 (29)	64.5 (12.3)	91.2 (12.3)	72.9 (17.2)
No	48.0	14.5	20.6	22.0	6.0	8.5	27.1
Not known	97.0	29.4	—	108.0	29.5	—	—

SD: standard deviation; IV: intravenous; NPSUM: National Programme on Substance Use Mortality.

a 1999–2019 excludes NFA *n* = 7, Usual address out of mainland U.K. *n* = 3, redacted *n* = 3; 2020–2024 excludes NFA *n* = 7, Usual address out of mainland U.K. *n* = 1.

## Discussion

This update on deaths following illicit ketamine use in England, Wales and Northern Ireland demonstrates an acceleration in both the raw number of deaths and the overall proportion of drug deaths where illicit ketamine was involved, a trend which is reflected in Scotland (National Records of Scotland, 2024). We also demonstrate that this increase is not just an artefact of the overall increasing trend in deaths following any drug use (ONS, 2024), as the proportion of all NPSUM deaths with illicit ketamine detections has also increased over the same time frame (2014: 0.9% of all NPSUM deaths; 2024: 3.9% of all NPSUM deaths). It also shows that the projected number of deaths expected to be received from 2019 in Corkery et al. (2021) proved accurate. This rise in mortality likely reflects a combination of expanding consumption (Home Office, 2025a) driven by the low cost of illicit ketamine (around £15–30 for 1 g compared to £80 for cocaine; Newcombe, 2024) in increasingly risky supply environments following the decline of the diverted pharmaceutical ketamine market and the subsequent growth of its unregulated clandestine production following its classification (and reclassification) under the Misuse of Drugs Act 1971. Recent findings from the Welsh Emerging Drugs and Identification of Novel Substances (WEDINOS) programme further support the presence of an active and intentional illicit ketamine market in the United Kingdom: between October 2024 and February 2025, 99.1% (*n* = 115/116) of samples submitted with the purchase intent of ketamine were confirmed to contain ketamine, with only two cases involving co-detected adulterants (WEDINOS, 2025). In contrast, ketamine was identified in just 0.9% (*n* = 32/3,420) of samples where another drug or combination of drugs had been the intended purchase in the same time period. These data highlight the reliability of illicit ketamine supply chains and reinforce the notion that illicit ketamine is being purposefully sought rather than incidentally consumed (WEDINOS, 2025). Taken together, these data support the growing public health concern posed by illicit ketamine use (Guerrini et al., 2025), and the need for assertive public health, harm reduction and treatment approaches.

Whilst there has been a concomitant increase in the number of illicit ketamine-related deaths, there has been a decrease over time in the proportion of deaths where it was detected *and* implicated. There was also an increase in the number of co-detected substances at post-mortem over time, and in the number of deaths where other substances, additional to illicit ketamine, were co-implicated in causing death. When taken together, these findings indicate that illicit ketamine is being increasingly used in complex polydrug settings where causality is either being shared with or completely attributed to other administered substances. Polydrug use is an increasingly common practice both in the United Kingdom and internationally (Boileau-Falardeau et al., 2022; Rock et al., 2022a, 2022b), with people intentionally administering multiple substances simultaneously or sequentially in order to enhance their respective effects or manage the symptoms of withdrawal (Boileau-Falardeau et al., 2022). The recent rise of ‘Tuci’ or ‘pink cocaine’ is demonstrative of this in the ketamine context as users are knowingly purchasing a product that contains a combination of substances – namely, illicit ketamine and MDMA (Palamar, 2023). Drug policies that target single substances can, therefore, often have limited effectiveness as they do not take into account the real-world context of substance use in a polydrug scenario (Crummy et al., 2020; EUDA, 2021): many fatal overdoses are due to synergistic effects between co-administered substances (Boileau-Falardeau et al., 2022), meaning policies focused on a single drug do not consider such interactions and associated differential risks; when a substance is subject to tighter controls, others that are equally or more dangerous – and often also poorly understood – can emerge, as has been observed with ‘street’ benzodiazepines (McAuley et al., 2022; Rock et al., 2025) and the synthetic opioid nitazenes (Holland et al., 2024); policies that control individual drugs are seldom accompanied by integrated harm reduction strategies (U.K. Government, 1971), such as supervised consumption or drug checking, that aim to mitigate risks of polydrug use (Holland et al., 2024). The control of single substances is, therefore, misaligned with what is often the reality of drug use, meaning the reclassification of ketamine under the Misuse of Drugs Act (U.K. Government, 1971) to a Class A substance may, therefore, not be effective in reducing its harms.

The pharmacoepidemiologic profile of illicit ketamine use has evolved over time to reflect broader trends in which substances once viewed as primarily recreational are increasingly embedded within complex, dependent, and riskier patterns of use driven by economic deprivation, housing instability, and entrenched marginalisation (Nutt et al., 2010). The increasing co-administration of other high-risk substances (e.g., opioids, benzodiazepines, cocaine, gabapentinoids) further indicates that illicit ketamine use is no longer confined to recreational scenes and is now also used to play a functional role within the self-regulation and harm-coping strategies employed by people who use drugs dependently (Nutt et al., 2010). Indeed, there are reports of individuals self-medicating with illicit ketamine for its analgesic properties to manage the pain associated with ketamine-induced bladder dysfunction, which perpetuates a painful cycle of injury and self-medication (Anderson et al., 2022). Recognising the proportional shift towards dependent illicit ketamine use is essential to inform appropriate harm reduction and treatment services, which must move beyond single-substance frameworks to address the realities of polydrug use within socioeconomically disadvantaged populations.

## Limitations

The NPSUM receives voluntary reports from coroners, so whilst the number of deaths following illicit ketamine use presented here is alarming, they are likely an underestimation. However, as the NPSUM receives reports from the majority of coronial jurisdictions across England, Wales and Northern Ireland, the trends observed are likely representative of the overall cases.

Whilst medical use of ketamine (and other drugs administered as part of medical treatment) is routinely reported to toxicologists to aid them in their interpretation of the toxicological findings, it is possible that this information was omitted/not made available to them. There may therefore be some cases with medically administered ketamine included in this study that we have identified as illicit in source.

Over time, toxicology screening libraries have expanded as new drugs have been introduced/emerged over the course of the study period, which have then been routinely tested for (e.g., the gabapentinoids gabapentin and pregabalin). It is therefore possible that part of the increase in the number of co-detected drugs may be reflecting these expanding screening libraries.

## Conclusions

The rising occurrence and context of deaths following illicit ketamine use underscores the need for drug policies and treatment services to more fully recognise the role illicit ketamine plays in complex, dependent drug use rather than viewing it solely through the lens of recreational use. A more comprehensive drug policy response is needed – rather than just a focus on the consideration of legislative change – to mitigate future deaths, including consideration of overdose prevention centres, expanded drug checking services, targeted education about the risks of polydrug use, better integration of ketamine use into treatment services and streamlining of referrals to clinical pathways. Furthermore, the evolving sociodemographic characteristics of illicit ketamine decedents highlight the importance of addressing structural determinants such as deprivation, unemployment and housing instability as part of any comprehensive drug policy response.
